# 2-(Tri­methyl­aza­nium­yl)ethyl hydrogen phosphate (phospho­choline) mono­hydrate

**DOI:** 10.1107/S160053681400779X

**Published:** 2014-04-12

**Authors:** Yohsuke Nikawa, Kyoko Fujita, Keiichi Noguchi, Hiroyuki Ohno

**Affiliations:** a Department of Biotechnology, Tokyo University of Agriculture and Technology, 2-24-16 Naka-cho, Koganei, Tokyo 184-8588, Japan; b Functional Ionic liquid Laboratories, Graduate School of Engineering, Tokyo University of Agriculture and Technology, 2-24-16, Naka-cho, Koganei, Japan; c Japan Science and Technology Agency (JST), Core Research for Evolutional Science and Technology (CREST), Chiyoda, Japan; d Instrumentation Analysis Center, Tokyo University of Agriculture and Technology, 2-24-16 Naka-cho, Koganei, Tokyo 184-8588, Japan

## Abstract

In the crystal structure of the title compound, C_5_H_14_NO_4_P·H_2_O, the zwitterionic phospho­choline mol­ecules are connected by an O—H⋯O hydrogen bond between the phosphate groups, forming a zigzag chain along the *b-*axis direction. The chains are further connected through O—H⋯O hydrogen bonds involving water mol­ecules, forming a layer parallel to (101). Three and one C—H⋯O inter­actions are also observed in the layer and between the layers, respectively. The conformation of the N—C—C—O backbone is *gauche* with a torsion angle of −75.8 (2)°

## Related literature   

For related structures, see: Fujita *et al.* (2009 ▶); Pearson & Pascher (1979 ▶); McAlister *et al.* (1979 ▶).
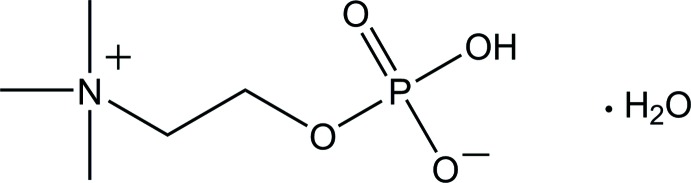


## Experimental   

### 

#### Crystal data  

C_5_H_14_NO_4_P·H_2_O
*M* *_r_* = 201.16
Monoclinic, 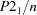

*a* = 10.4304 (2) Å
*b* = 6.8873 (1) Å
*c* = 13.4992 (3) Å
β = 105.800 (1)°
*V* = 933.11 (3) Å^3^
*Z* = 4
Cu *K*α radiation
μ = 2.59 mm^−1^
*T* = 193 K
0.60 × 0.40 × 0.40 mm

#### Data collection  

Rigaku R-AXIS RAPID diffractometer
Absorption correction: numerical (*NUMABS*; Rigaku, 1999 ▶) *T*_min_ = 0.306, *T*_max_ = 0.424
16036 measured reflections
1715 independent reflections
1632 reflections with *I* > 2σ(*I*)
*R*_int_ = 0.037

#### Refinement  

*R*[*F* ^2^ > 2σ(*F* ^2^)] = 0.038
*wR*(*F* ^2^) = 0.101
*S* = 1.13
1715 reflections
121 parameters
H atoms treated by a mixture of independent and constrained refinement
Δρ_max_ = 0.21 e Å^−3^
Δρ_min_ = −0.50 e Å^−3^

### 

Data collection: *PROCESS-AUTO* (Rigaku, 2004 ▶); cell refinement: *PROCESS-AUTO*; data reduction: *CrystalStructure* (Rigaku, 2010 ▶); program(s) used to solve structure: *Il Milione* (Burla *et al.*, 2007 ▶); program(s) used to refine structure: *SHELXL97* (Sheldrick, 2008 ▶); molecular graphics: *ORTEPIII* (Burnett & Johnson, 1996 ▶); software used to prepare material for publication: *SHELXL97*.

## Supplementary Material

Crystal structure: contains datablock(s) global, I. DOI: 10.1107/S160053681400779X/is5344sup1.cif

Structure factors: contains datablock(s) I. DOI: 10.1107/S160053681400779X/is5344Isup2.hkl

Supporting information file. DOI: 10.1107/S160053681400779X/is5344Isup3.cml

CCDC reference: 996006

Additional supporting information:  crystallographic information; 3D view; checkCIF report

## Figures and Tables

**Table 1:** Hydrogen-bond geometry (Å, °)

*D*—H⋯*A*	*D*—H	H⋯*A*	*D*⋯*A*	*D*—H⋯*A*
O2—H2*O*⋯O4^i^	0.80 (3)	1.74 (3)	2.525 (2)	167 (3)
O5—H5*OA*⋯O3^ii^	0.77 (3)	1.99 (3)	2.764 (2)	175 (3)
O5—H5*OB*⋯O3^iii^	0.75 (3)	2.04 (3)	2.784 (2)	172 (3)
C2—H2*A*⋯O3^iv^	0.99	2.47	3.440 (2)	167
C3—H3*B*⋯O5^v^	0.98	2.52	3.479 (3)	167
C3—H3*C*⋯O3^vi^	0.98	2.51	3.388 (2)	149
C5—H5*C*⋯O4^vii^	0.98	2.36	3.219 (3)	146

Symmetry codes: (i) 
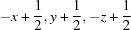
; (ii) 
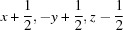
; (iii) 
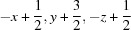
; (iv) 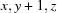; (v) 
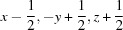
; (vi) 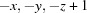; (vii) 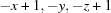.

**Fig. 1.**
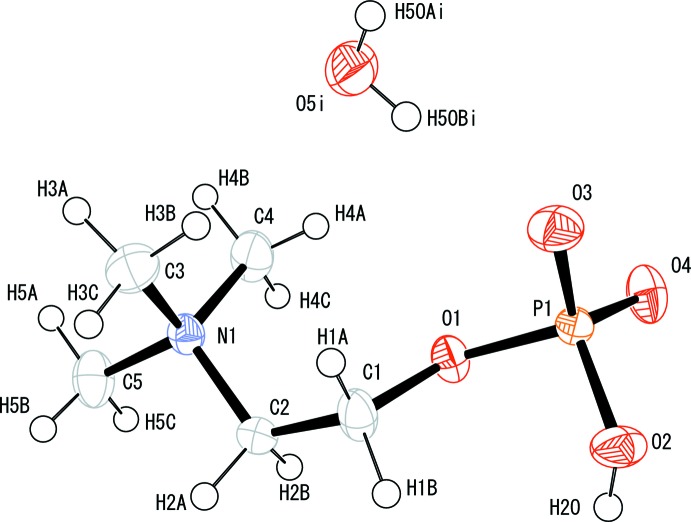
Displacement ellipsoid plot and atomic numbering scheme of the title compound. Ellipsoids are drawn at the 50% probability level and H atoms are shown as small spheres of arbitrary radii. [Symmetry code: (i) x - 1/2, -y + 1/2, z + 1/2.]

**Fig. 2.**
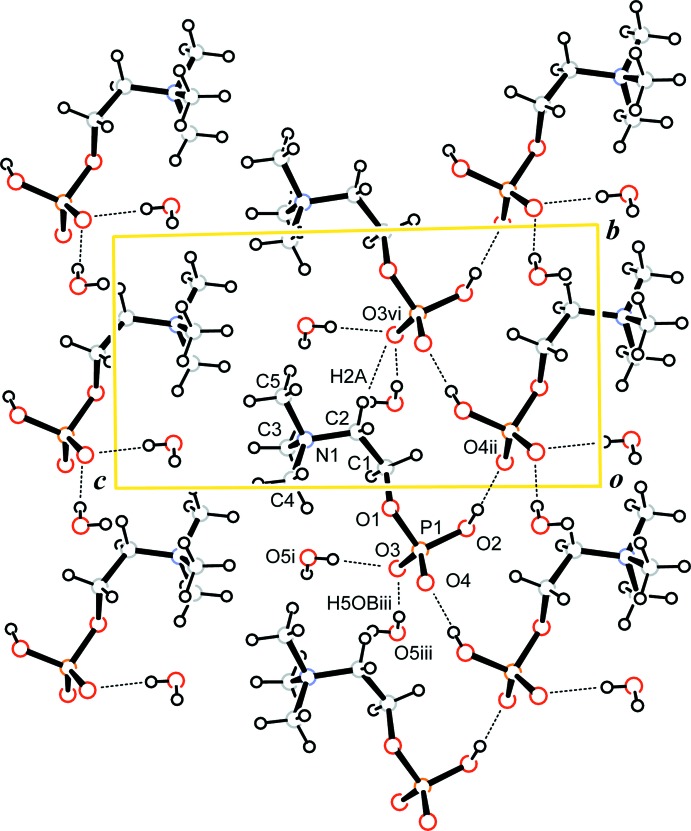
A packing diagram of the title compound, viewed along the a axis. Dashed lines indicate intermolecular O—H···O and C—H···O hydrogen bonds. [Symmetry codes: (i) x - 1/2, -y + 1/2, z + 1/2; (ii) -x + 1/2, y + 1/2, -z + 1/2; (iii) -x + 1/2, y - 3/2, -z + 1/2; (vi) x, 1 + y, z.]

**Fig. 3.**
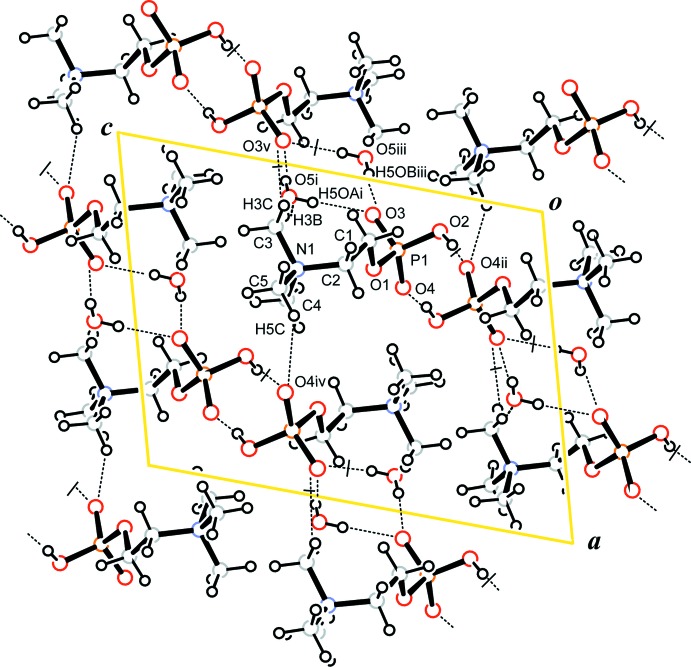
A packing diagram of the title compound, viewed along the b axis. Dashed lines indicate intermolecular O—H···O and C—H···O hydrogen bonds. [Symmetry codes: (i) x - 1/2, -y + 1/2, z + 1/2; (ii) -x + 1/2, y + 1/2, -z + 1/2; (iii) -x + 1/2, y - 3/2, -z + 1/2; (iv) 1 - x, -y, 1 - z; (v) -x, -y, 1 - z.]

## supplementary crystallographic information

### 1. Comment

Phosphocholine is similar to head groups of the phospholipid which is a major component of cell membranes. In past study, crystal structures of the compounds which have the phospholipid head group, such as 1,2-dimyristoyl-sn-*glycero*-3-phosphorylcholine dihydrate (Pearson & Pascher, 1979), choline phosphate calcium chloride tetrahydrate (McAlister *et al.*, 1979) and choline dihydrogen phosphate (Fujita *et al.*, 2009), were observed. We report herein the crystal structure of phosphocholine monohydrate.

The molecular structures of the title compound are shown in Fig. 1. The phosphate groups form the hydrogen bonds of O2···H—O4 linked to two neighboring phosphate groups (Fig. 2). These hydrogen bonds create a hydrogen bonding chain of phosphate groups along the *b* axis. In addition, phosphate groups are connected to the other neighboring phosphate group *via* two hydrogen bonds of O3···H—O5, with two water molecules (Fig. 3). Due to these hydrogen bonding network, molecules are arranged in layers parallel to the (101) plane. Four C—H···O interactions also occur in these layered structure (Table 1).

### 2. Experimental

Phosphorylcholine calcium chloride tetrahydrate was dissolved in water. The aqueous solution was treated on an anion exchange resin (Amberlite IRN77) and a cation exchange resin (TULSION-93). The solvent evaporated and the product was dried *in vacuo*. White powder was crystallized from a methanol solution. Acetonitrile was used as the antisolvent. This crystallization was repeated twice. Final purification was achieved by recrystallization from a saturated aqueous solution at room temperature for X-ray measurements.

The title compound was identified using ^1^H NMR, electrospray mass spectrometry, and elementary analysis. Spectroscopic analysis: ^1^H NMR (D_2_O, δ, p.p.m.): 3.214(s, 9H), 3.653(t, 2H), 4.286(m, 2H), HRMS(ESI) (m/*z*) calcd for C_5_H_14_NO_4_P [*M*+H]^+^ 184.0739, found 184.0849. Elementary analysis calculated for C_5_H_14_NO_4_P: C 32.79, H 7.71, N 7.65% found: C 32.22, H 7.383, N 8.017%.

### 3. Refinement

O-bound H atoms were located in a difference map and refined freely. H atoms of the CH_2_ and CH_3_ groups were subsequently refined as riding atoms, with C—H = 0.99 and 0.98 Å, respectively, and with *U*_iso_(H) = 1.2*U*_eq_(C).

### Crystal data

**Table:**

C_5_H_14_NO_4_P·H_2_O	*F*(000) = 432
*M**_r_* = 201.16	*D*_x_ = 1.432 Mg m^−^^3^
Monoclinic, *P*2_1_/*n*	Cu *K*α radiation, λ = 1.54187 Å
Hall symbol: -P 2yn	Cell parameters from 15511 reflections
*a* = 10.4304 (2) Å	θ = 3.4–68.2°
*b* = 6.8873 (1) Å	µ = 2.59 mm^−^^1^
*c* = 13.4992 (3) Å	*T* = 193 K
β = 105.800 (1)°	Block, colorless
*V* = 933.11 (3) Å^3^	0.60 × 0.40 × 0.40 mm
*Z* = 4	

### Data collection

**Table:**

Rigaku R-AXIS RAPID diffractometer	1715 independent reflections
Radiation source: rotating anode	1632 reflections with *I* > 2σ(*I*)
Graphite monochromator	*R*_int_ = 0.037
Detector resolution: 10.000 pixels mm^-1^	θ_max_ = 68.2°, θ_min_ = 4.8°
ω scans	*h* = −12→12
Absorption correction: numerical (*NUMABS*; Rigaku, 1999)	*k* = −8→8
*T*_min_ = 0.306, *T*_max_ = 0.424	*l* = −15→16
16036 measured reflections	

### Refinement

**Table:**

Refinement on *F*^2^	Primary atom site location: structure-invariant direct methods
Least-squares matrix: full	Secondary atom site location: difference Fourier map
*R*[*F*^2^ > 2σ(*F*^2^)] = 0.038	Hydrogen site location: inferred from neighbouring sites
*wR*(*F*^2^) = 0.101	H atoms treated by a mixture of independent and constrained refinement
*S* = 1.13	*w* = 1/[σ^2^(*F*_o_^2^) + (0.0594*P*)^2^ + 0.4329*P*] where *P* = (*F*_o_^2^ + 2*F*_c_^2^)/3
1715 reflections	(Δ/σ)_max_ < 0.001
121 parameters	Δρ_max_ = 0.21 e Å^−^^3^
0 restraints	Δρ_min_ = −0.50 e Å^−^^3^

### Special details

**Table:**

Geometry. All e.s.d.'s (except the e.s.d. in the dihedral angle between two l.s. planes) are estimated using the full covariance matrix. The cell e.s.d.'s are taken into account individually in the estimation of e.s.d.'s in distances, angles and torsion angles; correlations between e.s.d.'s in cell parameters are only used when they are defined by crystal symmetry. An approximate (isotropic) treatment of cell e.s.d.'s is used for estimating e.s.d.'s involving l.s. planes.
Refinement. Refinement of *F*^2^ against ALL reflections. The weighted *R*-factor *wR* and goodness of fit *S* are based on *F*^2^, conventional *R*-factors *R* are based on *F*, with *F* set to zero for negative *F*^2^. The threshold expression of *F*^2^ > σ(*F*^2^) is used only for calculating *R*-factors(gt) *etc*. and is not relevant to the choice of reflections for refinement. *R*-factors based on *F*^2^ are statistically about twice as large as those based on *F*, and *R*- factors based on ALL data will be even larger.

### Fractional atomic coordinates and isotropic or equivalent isotropic displacement parameters (Å^2^)

**Table:**

	*x*	*y*	*z*	*U*_iso_*/*U*_eq_	
P1	0.19372 (4)	−0.29328 (6)	0.34891 (3)	0.02017 (18)	
O1	0.26794 (11)	−0.11051 (17)	0.41531 (9)	0.0250 (3)	
O2	0.11276 (14)	−0.2045 (2)	0.24371 (10)	0.0340 (4)	
H2O	0.149 (3)	−0.124 (4)	0.219 (2)	0.058 (8)*	
O3	0.09520 (13)	−0.3768 (2)	0.39911 (10)	0.0316 (3)	
O4	0.30448 (13)	−0.4207 (2)	0.34008 (11)	0.0341 (3)	
N1	0.31816 (14)	0.1794 (2)	0.60363 (11)	0.0216 (3)	
C1	0.18974 (19)	0.0567 (3)	0.42416 (14)	0.0304 (4)	
H1A	0.1186	0.0186	0.4561	0.036*	
H1B	0.1469	0.1091	0.3547	0.036*	
C2	0.27544 (19)	0.2105 (2)	0.48844 (13)	0.0264 (4)	
H2A	0.2266	0.3353	0.4746	0.032*	
H2B	0.3567	0.2245	0.4646	0.032*	
C3	0.20152 (19)	0.1449 (3)	0.64541 (15)	0.0340 (4)	
H3A	0.2310	0.1464	0.7208	0.041*	
H3B	0.1619	0.0184	0.6217	0.041*	
H3C	0.1351	0.2473	0.6211	0.041*	
C4	0.4142 (2)	0.0135 (3)	0.63344 (15)	0.0367 (5)	
H4A	0.3717	−0.1062	0.6013	0.044*	
H4B	0.4401	−0.0015	0.7085	0.044*	
H4C	0.4936	0.0399	0.6100	0.044*	
C5	0.3865 (2)	0.3617 (3)	0.65134 (16)	0.0355 (5)	
H5A	0.4193	0.3447	0.7261	0.043*	
H5B	0.3232	0.4701	0.6361	0.043*	
H5C	0.4616	0.3896	0.6230	0.043*	
O5	0.58971 (16)	0.8315 (2)	0.10139 (12)	0.0367 (4)	
H5OA	0.593 (3)	0.838 (4)	0.045 (2)	0.043 (7)*	
H5OB	0.539 (3)	0.905 (4)	0.106 (2)	0.046 (8)*	

### Atomic displacement parameters (Å^2^)

**Table:**

	*U*^11^	*U*^22^	*U*^33^	*U*^12^	*U*^13^	*U*^23^
P1	0.0221 (3)	0.0191 (3)	0.0200 (3)	−0.00203 (15)	0.00695 (18)	−0.00011 (15)
O1	0.0229 (6)	0.0223 (6)	0.0273 (6)	0.0025 (5)	0.0025 (5)	−0.0048 (5)
O2	0.0380 (8)	0.0316 (8)	0.0263 (7)	−0.0155 (6)	−0.0015 (6)	0.0066 (5)
O3	0.0314 (7)	0.0330 (7)	0.0341 (7)	−0.0039 (6)	0.0151 (6)	0.0046 (6)
O4	0.0311 (7)	0.0313 (7)	0.0423 (8)	−0.0002 (6)	0.0141 (6)	−0.0127 (6)
N1	0.0195 (7)	0.0220 (7)	0.0227 (7)	0.0027 (5)	0.0046 (6)	−0.0014 (5)
C1	0.0308 (9)	0.0274 (10)	0.0276 (9)	0.0099 (8)	−0.0012 (7)	−0.0058 (7)
C2	0.0348 (10)	0.0208 (9)	0.0230 (9)	0.0030 (7)	0.0067 (7)	0.0025 (6)
C3	0.0297 (10)	0.0397 (11)	0.0357 (10)	−0.0025 (8)	0.0144 (8)	0.0026 (9)
C4	0.0380 (10)	0.0382 (11)	0.0292 (10)	0.0194 (9)	0.0013 (8)	−0.0007 (8)
C5	0.0308 (10)	0.0356 (11)	0.0397 (11)	−0.0083 (8)	0.0087 (8)	−0.0147 (9)
O5	0.0415 (9)	0.0374 (8)	0.0321 (8)	0.0114 (7)	0.0115 (7)	0.0050 (6)

### Geometric parameters (Å, º)

**Table:**

P1—O4	1.4812 (13)	C2—H2A	0.9900
P1—O3	1.4918 (13)	C2—H2B	0.9900
P1—O2	1.5655 (13)	C3—H3A	0.9800
P1—O1	1.6154 (12)	C3—H3B	0.9800
O1—C1	1.435 (2)	C3—H3C	0.9800
O2—H2O	0.79 (3)	C4—H4A	0.9800
N1—C3	1.493 (2)	C4—H4B	0.9800
N1—C5	1.500 (2)	C4—H4C	0.9800
N1—C4	1.501 (2)	C5—H5A	0.9800
N1—C2	1.512 (2)	C5—H5B	0.9800
C1—C2	1.501 (2)	C5—H5C	0.9800
C1—H1A	0.9900	O5—H5OA	0.77 (3)
C1—H1B	0.9900	O5—H5OB	0.74 (3)
			
O4—P1—O3	117.19 (8)	C1—C2—H2B	108.0
O4—P1—O2	113.47 (8)	N1—C2—H2B	108.0
O3—P1—O2	107.13 (8)	H2A—C2—H2B	107.2
O4—P1—O1	103.88 (7)	N1—C3—H3A	109.5
O3—P1—O1	109.49 (7)	N1—C3—H3B	109.5
O2—P1—O1	104.93 (7)	H3A—C3—H3B	109.5
C1—O1—P1	118.31 (10)	N1—C3—H3C	109.5
P1—O2—H2O	117 (2)	H3A—C3—H3C	109.5
C3—N1—C5	108.18 (14)	H3B—C3—H3C	109.5
C3—N1—C4	109.31 (15)	N1—C4—H4A	109.5
C5—N1—C4	108.53 (15)	N1—C4—H4B	109.5
C3—N1—C2	111.65 (13)	H4A—C4—H4B	109.5
C5—N1—C2	107.20 (14)	N1—C4—H4C	109.5
C4—N1—C2	111.84 (14)	H4A—C4—H4C	109.5
O1—C1—C2	110.62 (14)	H4B—C4—H4C	109.5
O1—C1—H1A	109.5	N1—C5—H5A	109.5
C2—C1—H1A	109.5	N1—C5—H5B	109.5
O1—C1—H1B	109.5	H5A—C5—H5B	109.5
C2—C1—H1B	109.5	N1—C5—H5C	109.5
H1A—C1—H1B	108.1	H5A—C5—H5C	109.5
C1—C2—N1	117.20 (15)	H5B—C5—H5C	109.5
C1—C2—H2A	108.0	H5OA—O5—H5OB	105 (3)
N1—C2—H2A	108.0		
			
O4—P1—O1—C1	170.10 (13)	O1—C1—C2—N1	−75.8 (2)
O3—P1—O1—C1	−63.97 (14)	C3—N1—C2—C1	−54.4 (2)
O2—P1—O1—C1	50.72 (15)	C5—N1—C2—C1	−172.72 (15)
P1—O1—C1—C2	179.94 (12)	C4—N1—C2—C1	68.4 (2)

### Hydrogen-bond geometry (Å, º)

**Table:**

*D*—H···*A*	*D*—H	H···*A*	*D*···*A*	*D*—H···*A*
O2—H2*O*···O4^i^	0.80 (3)	1.74 (3)	2.525 (2)	167 (3)
O5—H5*OA*···O3^ii^	0.77 (3)	1.99 (3)	2.764 (2)	175 (3)
O5—H5*OB*···O3^iii^	0.75 (3)	2.04 (3)	2.784 (2)	172 (3)
C2—H2*A*···O3^iv^	0.99	2.47	3.440 (2)	167
C3—H3*B*···O5^v^	0.98	2.52	3.479 (3)	167
C3—H3*C*···O3^vi^	0.98	2.51	3.388 (2)	149
C5—H5*C*···O4^vii^	0.98	2.36	3.219 (3)	146

Symmetry codes: (i) −*x*+1/2, *y*+1/2, −*z*+1/2; (ii) *x*+1/2, −*y*+1/2, *z*−1/2; (iii) −*x*+1/2, *y*+3/2, −*z*+1/2; (iv) *x*, *y*+1, *z*; (v) *x*−1/2, −*y*+1/2, *z*+1/2; (vi) −*x*, −*y*, −*z*+1; (vii) −*x*+1, −*y*, −*z*+1.
